# Mechanism of Dayuanyin in the treatment of coronavirus disease 2019 based on network pharmacology and molecular docking

**DOI:** 10.1186/s13020-020-00346-6

**Published:** 2020-06-12

**Authors:** Xiaofeng Ruan, Peng Du, Kang Zhao, Jucun Huang, Hongmei Xia, Dan Dai, Shu Huang, Xiang Cui, Liming Liu, Jianjun Zhang

**Affiliations:** 1 College of Traditional Chinese Medicine, Hubei University of Traditional Chinese Medicine, Wuhan, 430070 China; 2 Department of Rehabilitation Medicine, Xiangyang Central Hospital, Xiangyang, 441021 Hubei China; 3 Department of Liver Medicine, Hubei NO.3 People’s Hospital of Jianghan University, Wuhan, 430033 China; 4 Department of Liver Medicine, AnKang Hospital of Traditional Chinese Medicine, Ankang, 72500 Shaanxi China

**Keywords:** Dayuanyin, Coronavirus disease 2019, Network pharmacology, Molecular docking, Mechanism research

## Abstract

**Background:**

At present, coronavirus disease 2019 (COVID-19), caused by infection with severe acute respiratory syndrome coronavirus 2, is spreading all over the world, with disastrous consequences for people of all countries. The traditional Chinese medicine prescription Dayuanyin (DYY), a classic prescription for the treatment of plague, has shown significant effects in the treatment of COVID-19. However, its specific mechanism of action has not yet been clarified. This study aims to explore the mechanism of action of DYY in the treatment of COVID-19 with the hope of providing a theoretical basis for its clinical application.

**Methods:**

First, the TCMSP database was searched to screen the active ingredients and corresponding target genes of the DYY prescription and to further identify the core compounds in the active ingredient. Simultaneously, the Genecards database was searched to identify targets related to COVID-19. Then, the STRING database was applied to analyse protein–protein interaction, and Cytoscape software was used to draw a network diagram. The R language and DAVID database were used to analyse GO biological processes and KEGG pathway enrichment. Second, AutoDock Vina and other software were used for molecular docking of core targets and core compounds. Finally, before and after application of DYY, the core target gene IL6 of COVID-19 patients was detected by ELISA to validate the clinical effects.

**Results:**

First, 174 compounds, 7053 target genes of DYY and 251 genes related to COVID-19 were selected, among which there were 45 target genes of DYY associated with treatment of COVID-19. This study demonstrated that the use of DYY in the treatment of COVID-19 involved a variety of biological processes, and DYY acted on key targets such as IL6, ILIB, and CCL2 through signaling pathways such as the IL-17 signaling pathway, AGE-RAGE signaling pathway in diabetic complications, and cytokine–cytokine receptor interaction. DYY might play a vital role in treating COVID-19 by suppressing the inflammatory storm and regulating immune function. Second, the molecular docking results showed that there was a certain affinity between the core compounds (kaempferol, quercetin, 7-Methoxy-2-methyl isoflavone, naringenin, formononetin) and core target genes (IL6, IL1B, CCL2). Finally, clinical studies showed that the level of IL6 was elevated in COVID-19 patients, and DYY can reduce its levels.

**Conclusions:**

DYY may treat COVID-19 through multiple targets, multiple channels, and multiple pathways and is worthy of clinical application and promotion.

## Background

Coronavirus disease 2019 (COVID-19) was first discovered in Wuhan, China, on December 12, 2019, but to date, no definitive conclusion has been drawn about its origin. According to the classification of syndromes in traditional Chinese medicine, COVID-19 is classified as an “epidemic disease” (damp-warm disease), and it is a highly contagious disease. In the early stages of damp-warm diseases, “damp-warm disease with syndrome of pathogen blocking pleuro-diaphragmatic interspace” is very common and is a specific stage and phenomenon in the pathological process of the disease. This symptom first appeared in the *Theory of Epidemic Febrile Disease* by Wu Youke during the Ming Dynasty, and he created Dayuanyin (DYY), described in the book. After 2019-nCoV invades the human body, it disturbs and damages the human immune system, further causing different degrees of damage to various organs throughout the body [1, 2]. DYY, a traditional Chinese medicine prescription, has played an important role in the prevention and treatment of epidemic diseases in documented history and literature. It has been used to treat influenza [3], atypical pneumonia [4], AIDS [5] and other diseases and has proven to be very effective in clinical applications. At the same time, through clinical observation of COVID-19 patients in the early stage of DYY treatment, it was found that this prescription can improve the clinical symptoms and signs of patients, improve the prognosis of patients, and shorten the course of disease [6, 7], making it worthy of clinical application and promotion. However, its mechanism of action in COVID-19 patients has not yet been clarified.

Network pharmacology, originally proposed by Andrew L Hopkins, includes systems biology, pharmacology, mathematics, computer network analysis, etc. As a useful tool for systematically evaluating and demonstrating the rationality of drugs, it has now been widely accepted [8, 9]. The application of network pharmacology in traditional Chinese medicine provides us with new possibilities for screening active ingredients of drugs and targets for disease treatment, which is helpful for explaining the mechanism of action of drugs for disease treatment at a system level [10]. Molecular docking is a theoretical simulation method that mainly studies intermolecular interactions and predicts their binding mode and affinity [11]. Not only can it be used for drug development, but it can also provide keen insights into protein function prediction and other important issues [12].

**Fig. 1:**
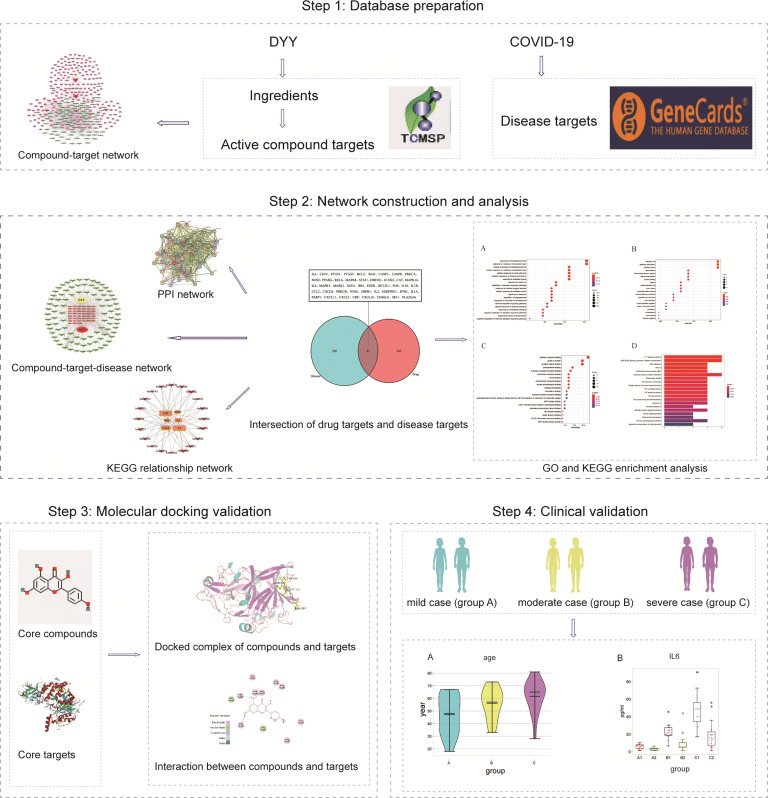
Technological road-map

This study aimed to use network pharmacology and molecular docking to preliminarily explore the mechanism of action of this prescription in the treatment of COVID-19 patients, with the goal of widely using this prescription for COVID-19 patients with early damp-warm syndromes to improve the patients’ condition and to prevent the ongoing COVID-19 outbreak. A technological road-map of the experimental procedures of our study is shown in Fig. 1.

## Methods

### Acquisition of the chemical composition and target information of DYY

The Traditional Chinese Medicine Systems Pharmacology Database and Analysis Platform (TCMSP) records 499 common traditional Chinese medicines (Chinese Pharmacopoeia 2010 edition) and elaborates their ingredients, the corresponding target information and common disease information related to traditional Chinese medicines [13]. The database provides pharmacokinetic information for each compound, such as drug-like (DL), oral bioavailability (OB), and blood-brain barrier (BBB). In this study, the TCMSP database (http://tcmspw.com/tcmsp.php) was used to search for and determine the active ingredients in the composition of the DYY decoction. At the same time, target genes were predicted for these active ingredients. OB and DL property are important reference standards for evaluating whether compounds can be used as drugs. In this study, OB ≥ 30% and DL ≥ 0.18 were used as screening thresholds [14]. According to the selected active ingredients of DYY, the target genes corresponding to the above active ingredients derived from DrugBank were further screened using Perl language in combination with the TCMSP database.

### Gene name standardization

Perl language was used in combination with the UniProtKB search function in the UniProt database (http://www.uniprot.org/, update in 2018-04-10), the protein name was entered, the species was limited to humans, and the retrieved protein name was corrected to the official name of the protein.

### Construction of network diagrams of compounds and corresponding targets

The compounds and predicted targets in the DYY formula obtained through the TCMSP database were imported into Cytoscape 3.6.1 software, and a compound-target network diagram was drawn to obtain the top five core compounds.

### Acquisition of disease targets

The keyword “novel coronavirus pneumonia” was entered into the Genecards (https://www.genecards.org/, version 4.12) database to obtain target genes related to the COVID-19 disease.

### Intersection of disease genes and drug genes

The target genes predicted from the active ingredients in DYY were intersected and mapped with the target genes predicted for the COVID-19 disease to obtain the target genes of DYY for the treatment of COVID-19. The Venn Diagram package in R was used to draw a Venn diagram.

### Protein–protein interaction analysis and core target screening

The target genes for DYY treatment of COVID-19 were entered into the STRING database for protein–protein interaction (PPI) analysis, “Homo sapiens” was selected, the minimum required interaction score was set to > 0.9, the protein interaction network map was downloaded, and R3.5.0 was used to screen core genes.

### Construction of network visualization

The active ingredients of DYY, the targets corresponding to the active ingredients, and the targets predicted for the COVID-19 disease were imported into Cytoscape 3.6.1 software, and a drug-target-disease network diagram was constructed for network visualization.

### GO analysis and KEGG pathway enrichment analysis

The DAVID database (http://david.abcc.ncifcrf.gov/) can functionally annotate many genes and help us understand the biological process and meaning behind genes. The target genes selected above were combined with R language and DAVID database for Gene Ontology (GO) biological process enrichment and Kyoto Encyclopedia of Genes and Genomes (KEGG) pathway enrichment.

### Construction of KEGG relationship network

The pathway ID numbers and the genes involved in the KEGG-enriched pathways were imported into Cytoscape software, the number of adjacent nodes in the network was calculated, and the size of the nodes in the network was determined according to the number of adjacent nodes to construct a KEGG relationship network.

### Molecular docking verification of core compounds and core target genes

Firstly, the top five core compounds were selected, and the two-dimensional structure diagrams of the compounds were downloaded from the PubChem database, imported into Chem3D software to draw the three-dimensional structure diagrams of the core compounds and optimize the energy, and saved in mol2 format. Then, the files were imported into AutoDockTools-1.5.6 software to add charge and display rotatable keys and then saved in pdbqt format. Secondly, the protein crystal structures corresponding to the core target genes were downloaded from the PDB database, imported into Pymol software to remove water molecules and heteromolecules, imported into AutoDockTools-1.5.6 software to add hydrogen atoms and charge operations, saved to pdbqt format, and imported into Discovery Studio 3.5 Client software to search for active pockets. Finally, the above core compounds were used as ligands, and the proteins corresponding to the core target genes were used as receptors for molecular docking. The results were analysed and interpreted using PyMOL software and Discovery Studio 3.5 Client.

### Clinical validation of the core target IL6

In this study, a total of 45 patients who were hospitalized in Third People’s Hospital of Hubei Province and Lei Shen Shan Hospital during the period from January 25, 2019 to March 8, 2020 were selected. The TCM syndrome of the selected patients was “plague (syndrome of pathogen hidden in interpleuro-diaphramatic space)”, and DYY was used for treatment. ELISA was used to detect changes in IL6 levels at the time of before and after treatment for 1 week. All statistical analyses were performed by GraphPad Prism version 7.00 software. T test was used for comparisons before and after treatment in each group. Our data are expressed as the mean ± standard deviation (SD). A value of *p *< 0.05 was considered significant.

### Mechanism of action of DYY in the treatment of COVID-19

Adobe Illustrator CC software was used to draw the chart for the specific mechanism of DYY treatment of COVID-19.

## Results

### Acquisition of the active ingredient and target information of DYY

The composition of DYY is magnolia officinalis(MO), amomum(AM), arecae semen(AS), herbaceous peony(HP), scutellariae radix(SR), anemarrhenae rhizoma(AR) and licorice(LR), as shown in Additional file 1: Fig. S1. A total of 839 DYY active ingredients were obtained from TCMSP database. Among them, the number of active ingredients from magnolia officinalis, amomum, arecae semen, herbaceous peony, scutellariae radix, anemarrhenae rhizoma and licorice was 52, 139, 59, 85, 143, 81, 280, respectively. After screening by the ADME standard (OB ≥ 30%, DL ≥ 0.18), 174 compounds were obtained, among which the number of compound from magnolia officinalis, amomum, arecae semen, herbaceous peony, scutellariae radix, anemarrhenae rhizoma and licorice was 8, 2, 8, 13, 3, 15, 92, respectively. Among them, MOL000073 (ent-Epicatechin) was a common compound of amomum, scutellariae radix, and licorice; MOL004961 (quercetin) was a common compound of licorice and arecae semen; MOL000211 (mairin) was a common compound of herbaceous peony and licorice; MOL000358 (beta-sitosterol) was a common compound of scutellariae radix and herbaceous peony; MOL000359 (sitosterol) was a common compound of licorice, scutellariae radix and herbaceous peony; and MOL000449 (stigmasterol) was a common compound of anemarrhenae rhizoma and scutellariae radix.

According to the results obtained by screening the active ingredients against the TCMSP database, there were a total of 7053 targets in the DrugBank. Among them, the number of targets of magnolia officinalis, amomum, arecae semen, herbaceous peony, scutellariae radix, anemarrhenae rhizoma and licorice was 379,1162,406, 990, 1203, 407 and 2506, respectively. After screening by the ADME standard (OB ≥ 30%, DL ≥ 0.18), 2766 targets related to the bioactive components were obtained, among which the number of targets from magnolia officinalis, amomum, arecae semen, herbaceous peony, scutellariae radix, anemarrhenae rhizoma and licorice was 32, 179, 41, 122, 436, 188, 1768, respectively. The distribution of candidate compounds and targets in each herb is shown in Table 1.

**Table 1:** Active ingredients of compounds

MOL ID	Component name	OB%	DL	Number of targets	Herb
MOL010482	WLN: 6OVR BVO6	43.74	0.24	9	AS
MOL010485	EPA	45.66	0.21	2	AS
MOL010489	Resivit	30.84	0.27	4	AS
MOL001749	ZINC03860434	43.59	0.35	4	AS
MOL002032	DNOP	40.59	0.4	5	AS
MOL002372	(6Z,10E,14E,18E)-2,6,10,15,19,23-hexamethyltetracosa-2,6,10,14,18,22-hexaene	33.55	0.42	0	AS
MOL000004	Procyanidin B1	67.87	0.66	11	AS
MOL000073	ent-Epicatechin	48.96	0.24	6	AS
MOL000073	ent-Epicatechin	48.96	0.24	6	AM
MOL000074	(4E,6E)-1,7-bis(4-hydroxyphenyl)hepta-4,6-dien-3-one	67.92	0.24	7	AM
MOL000085	Beta-daucosterol_qt	36.91	0.75	1	AM
MOL000088	Beta-sitosterol 3-O-glucoside_qt	36.91	0.75	0	AM
MOL000092	Daucosterin_qt	36.91	0.76	0	AM
MOL000094	Daucosterol_qt	36.91	0.76	0	AM
MOL000096	(−)-Catechin	49.68	0.24	11	AM
MOL000098	quercetin	46.43	0.28	154	AM
MOL005970	Eucalyptol	60.62	0.32	25	MO
MOL005980	Neohesperidin	57.44	0.27	7	MO
MOL001910	11alpha,12alpha-epoxy-3beta-23-dihydroxy-30-norolean-20-en-28,12beta-olide	64.77	0.38	0	HP
MOL001918	Paeoniflorgenone	87.59	0.37	0	HP
MOL001919	(3S,5R,8R,9R,10S,14S)-3,17-dihydroxy-4,4,8,10,14-pentamethyl-2,3,5,6,7,9-hexahydro-1H-cyclopenta[a]phenanthrene-15,16-dione	43.56	0.53	2	HP
MOL001921	Lactiflorin	49.12	0.8	0	HP
MOL001924	Paeoniflorin	53.87	0.79	4	HP
MOL001925	Paeoniflorin_qt	68.18	0.4	0	HP
MOL001928	Albiflorin_qt	66.64	0.33	0	HP
MOL001930	Benzoyl paeoniflorin	31.27	0.75	0	HP
MOL000211	Mairin	55.38	0.78	1	HP
MOL000358	Beta-sitosterol	36.91	0.75	38	HP
MOL000359	Sitosterol	36.91	0.75	3	HP
MOL000422	Kaempferol	41.88	0.24	63	HP
MOL000492	(+)-Catechin	54.83	0.24	11	HP
MOL001689	Acacetin	34.97	0.24	0	SR
MOL000173	Wogonin	30.68	0.23	0	SR
MOL000228	(2R)-7-hydroxy-5-methoxy-2-phenylchroman-4-one	55.23	0.2	22	SR
MOL002714	Baicalein	33.52	0.21	37	SR
MOL002908	5,8,2′-Trihydroxy-7-methoxyflavone	37.01	0.27	0	SR
MOL002909	5,7,2,5-tetrahydroxy-8,6-dimethoxyflavone	33.82	0.45	13	SR
MOL002910	Carthamidin	41.15	0.24	4	SR
MOL002911	2,6,2′,4′-tetrahydroxy-6′-methoxychaleone	69.04	0.22	0	SR
MOL002913	Dihydrobaicalin_qt	40.04	0.21	4	SR
MOL002914	Eriodyctiol (flavanone)	41.35	0.24	8	SR
MOL002915	Salvigenin	49.07	0.33	18	SR
MOL002917	5,2′,6′-Trihydroxy-7,8-dimethoxyflavone	45.05	0.33	17	SR
MOL002925	5,7,2′,6′-Tetrahydroxyflavone	37.01	0.24	6	SR
MOL002926	Dihydrooroxylin A	38.72	0.23	0	SR
MOL002927	Skullcapflavone II	69.51	0.44	21	SR
MOL002928	Oroxylin a	41.37	0.23	26	SR
MOL002932	Panicolin	76.26	0.29	14	SR
MOL002933	5,7,4′-Trihydroxy-8-methoxyflavone	36.56	0.27	18	SR
MOL002934	NEOBAICALEIN	104.34	0.44	22	SR
MOL002937	DIHYDROOROXYLIN	66.06	0.23	11	SR
MOL000358	beta-sitosterol	36.91	0.75	38	SR
MOL000359	Sitosterol	36.91	0.75	3	SR
MOL000525	Norwogonin	39.4	0.21	12	SR
MOL000552	5,2′-Dihydroxy-6,7,8-trimethoxyflavone	31.71	0.35	21	SR
MOL000073	ent-Epicatechin	48.96	0.24	6	SR
MOL000449	Stigmasterol	43.83	0.76	31	SR
MOL001458	Coptisine	30.67	0.86	9	SR
MOL001490	bis[(2S)-2-ethylhexyl] benzene-1,2-dicarboxylate	43.59	0.35	1	SR
MOL001506	Supraene	33.55	0.42	0	SR
MOL002879	Diop	43.59	0.39	3	SR
MOL002897	Epiberberine	43.09	0.78	11	SR
MOL008206	Moslosooflavone	44.09	0.25	25	SR
MOL010415	11,13-Eicosadienoic acid, methyl ester	39.28	0.23	1	SR
MOL012245	5,7,4′-trihydroxy-6-methoxyflavanone	36.63	0.27	6	SR
MOL012246	5,7,4′-trihydroxy-8-methoxyflavanone	74.24	0.26	6	SR
MOL012266	Rivularin	37.94	0.37	22	SR
MOL001677	Asperglaucide	58.02	0.52	5	AR
MOL003773	Mangiferolic acid	36.16	0.84	0	AR
MOL000422	Kaempferol	41.88	0.24	63	AR
MOL004373	Anhydroicaritin	45.41	0.44	37	AR
MOL004489	Anemarsaponin F_qt	60.06	0.79	1	AR
MOL004492	Chrysanthemaxanthin	38.72	0.58	0	AR
MOL004497	Hippeastrine	51.65	0.62	11	AR
MOL004514	Timosaponin B III_qt	35.26	0.87	2	AR
MOL000449	Stigmasterol	43.83	0.76	31	AR
MOL004528	Icariin I	41.58	0.61	1	AR
MOL004540	Anemarsaponin C_qt	35.5	0.87	3	AR
MOL004542	Anemarsaponin E_qt	30.67	0.86	0	AR
MOL000483	(Z)-3-(4-hydroxy-3-methoxy-phenyl)-N-[2-(4-hydroxyphenyl)ethyl]acrylamide	118.35	0.26	8	AR
MOL000546	diosgenin	80.88	0.81	16	AR
MOL000631	Coumaroyltyramine	112.9	0.2	10	AR
MOL001484	Inermine	75.18	0.54	17	LR
MOL001792	DFV	32.76	0.18	12	LR
MOL000211	Mairin	55.38	0.78	1	LR
MOL002311	Glycyrol	90.78	0.67	11	LR
MOL000239	Jaranol	50.83	0.29	13	LR
MOL002565	Medicarpin	49.22	0.34	34	LR
MOL000354	Isorhamnetin	49.6	0.31	37	LR
MOL000359	Sitosterol	36.91	0.75	3	LR
MOL003656	Lupiwighteone	51.64	0.37	21	LR
MOL003896	7-Methoxy-2-methyl isoflavone	42.56	0.2	43	LR
MOL000392	Formononetin	69.67	0.21	39	LR
MOL000417	Calycosin	47.75	0.24	22	LR
MOL000422	Kaempferol	41.88	0.24	63	LR
MOL004328	Naringenin	59.29	0.21	37	LR
MOL004805	(2S)-2-[4-hydroxy-3-(3-methylbut-2-enyl)phenyl]-8,8-dimethyl-2,3-dihydropyrano[2,3-f]chromen-4-one	31.79	0.72	12	LR
MOL004806	Euchrenone	30.29	0.57	10	LR
MOL004808	Glyasperin B	65.22	0.44	21	LR
MOL004810	Glyasperin F	75.84	0.54	18	LR
MOL004811	Glyasperin C	45.56	0.4	24	LR
MOL004814	Isotrifoliol	31.94	0.42	14	LR
MOL004815	(E)-1-(2,4-dihydroxyphenyl)-3-(2,2-dimethylchromen-6-yl)prop-2-en-1-one	39.62	0.35	20	LR
MOL004820	Kanzonols W	50.48	0.52	21	LR
MOL004824	(2S)-6-(2,4-dihydroxyphenyl)-2-(2-hydroxypropan-2-yl)-4-methoxy-2,3-dihydrofuro[3,2-g]chromen-7-one	60.25	0.63	21	LR
MOL004827	Semilicoisoflavone B	48.78	0.55	17	LR
MOL004828	Glepidotin A	44.72	0.35	25	LR
MOL004829	Glepidotin B	64.46	0.34	15	LR
MOL004833	Phaseolinisoflavan	32.01	0.45	22	LR
MOL004835	Glypallichalcone	61.6	0.19	27	LR
MOL004838	8-(6-hydroxy-2-benzofuranyl)-2,2-dimethyl-5-chromenol	58.44	0.38	6	LR
MOL004841	Licochalcone B	76.76	0.19	19	LR
MOL004848	Licochalcone G	49.25	0.32	17	LR
MOL004849	3-(2,4-dihydroxyphenyl)-8-(1,1-dimethylprop-2-enyl)-7-hydroxy-5-methoxy-coumarin	59.62	0.43	23	LR
MOL004855	Licoricone	63.58	0.47	15	LR
MOL004856	Gancaonin A	51.08	0.4	20	LR
MOL004857	Gancaonin B	48.79	0.45	22	LR
MOL004860	licorice glycoside E	32.89	0.27	0	LR
MOL004863	3-(3,4-dihydroxyphenyl)-5,7-dihydroxy-8-(3-methylbut-2-enyl)chromone	66.37	0.41	18	LR
MOL004864	5,7-dihydroxy-3-(4-methoxyphenyl)-8-(3-methylbut-2-enyl)chromone	30.49	0.41	20	LR
MOL004866	2-(3,4-dihydroxyphenyl)-5,7-dihydroxy-6-(3-methylbut-2-enyl)chromone	44.15	0.41	16	LR
MOL004879	Glycyrin	52.61	0.47	17	LR
MOL004882	Licocoumarone	33.21	0.36	7	LR
MOL004883	Licoisoflavone	41.61	0.42	19	LR
MOL004884	Licoisoflavone B	38.93	0.55	17	LR
MOL004885	Licoisoflavanone	52.47	0.54	20	LR
MOL004891	Shinpterocarpin	80.3	0.73	30	LR
MOL004898	(E)-3-[3,4-dihydroxy-5-(3-methylbut-2-enyl)phenyl]-1-(2,4-dihydroxyphenyl)prop-2-en-1-one	46.27	0.31	12	LR
MOL004903	Liquiritin	65.69	0.74	6	LR
MOL004904	Licopyranocoumarin	80.36	0.65	16	LR
MOL004905	3,22-Dihydroxy-11-oxo-delta(12)-oleanene-27-alpha-methoxycarbonyl-29-oic acid	34.32	0.55	0	LR
MOL004907	Glyzaglabrin	61.07	0.35	18	LR
MOL004908	Glabridin	53.25	0.47	25	LR
MOL004910	Glabranin	52.9	0.31	11	LR
MOL004911	Glabrene	46.27	0.44	19	LR
MOL004912	Glabrone	52.51	0.5	21	LR
MOL004913	1,3-dihydroxy-9-methoxy-6-benzofurano[3,2-c]chromenone	48.14	0.43	10	LR
MOL004914	1,3-dihydroxy-8,9-dimethoxy-6-benzofurano[3,2-c]chromenone	62.9	0.53	9	LR
MOL004915	Eurycarpin A	43.28	0.37	19	LR
MOL004917	Glycyroside	37.25	0.79	0	LR
MOL004924	(-)-Medicocarpin	40.99	0.95	2	LR
MOL004935	Sigmoidin-B	34.88	0.41	6	LR
MOL004941	(2R)-7-hydroxy-2-(4-hydroxyphenyl)chroman-4-one	71.12	0.18	15	LR
MOL004945	(2S)-7-hydroxy-2-(4-hydroxyphenyl)-8-(3-methylbut-2-enyl)chroman-4-one	36.57	0.32	12	LR
MOL004948	Isoglycyrol	44.7	0.84	7	LR
MOL004949	Isolicoflavonol	45.17	0.42	15	LR
MOL004957	HMO	38.37	0.21	27	LR
MOL004959	1-Methoxyphaseollidin	69.98	0.64	29	LR
MOL004961	Quercetin der.	46.45	0.33	17	LR
MOL004966	3′-Hydroxy-4′-O-Methylglabridin	43.71	0.57	28	LR
MOL000497	Licochalcone a	40.79	0.29	32	LR
MOL004974	3′-Methoxyglabridin	46.16	0.57	28	LR
MOL004978	2-[(3R)-8,8-dimethyl-3,4-dihydro-2H-pyrano[6,5-f]chromen-3-yl]-5-methoxyphenol	36.21	0.52	31	LR
MOL004980	Inflacoumarin A	39.71	0.33	15	LR
MOL004985	icos-5-enoic acid	30.7	0.2	1	LR
MOL004988	Kanzonol F	32.47	0.89	8	LR
MOL004989	6-prenylated Eriodictyol	39.22	0.41	8	LR
MOL004990	7,2′,4′-trihydroxy-5-methoxy-3-arylcoumarin	83.71	0.27	15	LR
MOL004991	7-Acetoxy-2-methylisoflavone	38.92	0.26	25	LR
MOL004993	8-prenylated eriodictyol	53.79	0.4	8	LR
MOL004996	Gadelaidic acid	30.7	0.2	1	LR
MOL000500	Vestitol	74.66	0.21	30	LR
MOL005000	Gancaonin G	60.44	0.39	20	LR
MOL005001	Gancaonin H	50.1	0.78	12	LR
MOL005003	Licoagrocarpin	58.81	0.58	29	LR
MOL005007	Glyasperins M	72.67	0.59	26	LR
MOL005008	Glycyrrhiza flavonol A	41.28	0.6	17	LR
MOL005012	Licoagroisoflavone	57.28	0.49	18	LR
MOL005013	18α-hydroxyglycyrrhetic acid	41.16	0.71	0	LR
MOL005016	Odoratin	49.95	0.3	20	LR
MOL005017	Phaseol	78.77	0.58	14	LR
MOL005018	Xambioona	54.85	0.87	8	LR
MOL005020	Dehydroglyasperins C	53.82	0.37	18	LR
MOL000098	Quercetin	46.43	0.28	154	LR

### Construction of network diagrams of compounds and corresponding targets

**Fig. 2:**
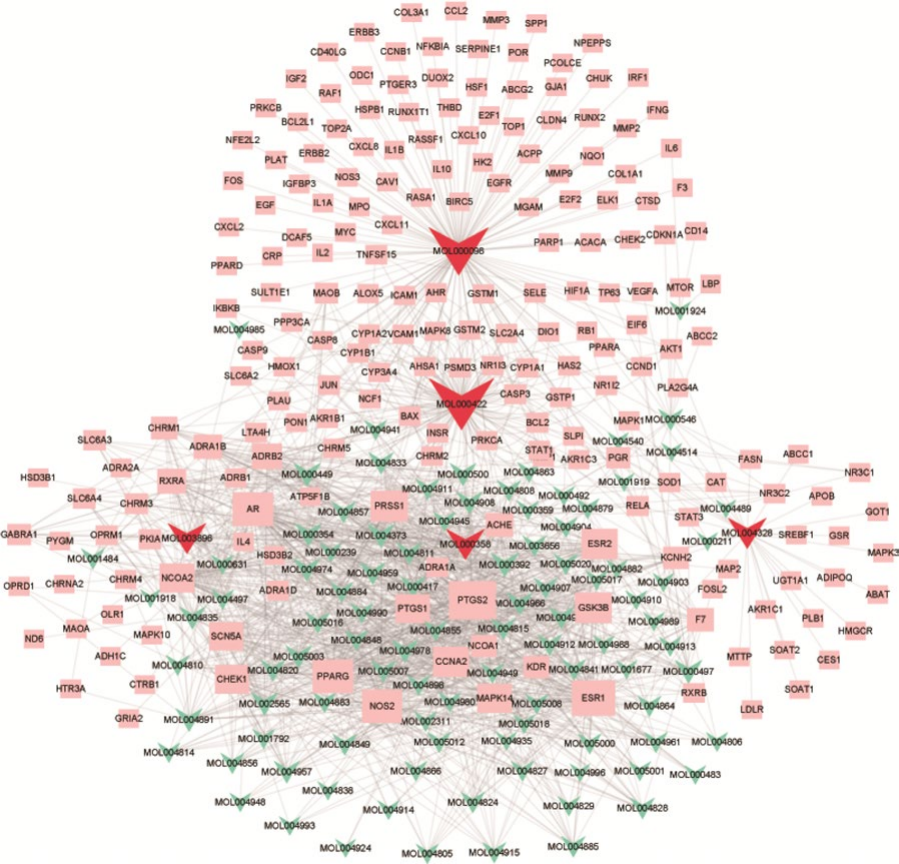
Compounds and corresponding targets network diagram. The green arrows in the figure represent the MOL numbers of the compound, and the red arrows represent the top five compounds with the largest area. The pink rectangles represent the target genes predicted by the compound. Lines represent the relationship between nodes. The larger the graph area is, the more connections there are to the node, and the more important the node is

The compounds and corresponding targets in the DYY formula were imported into Cytoscape software to draw a compound-target network diagram (see Fig. 2). In this study, degree was selected as a measure of node importance. With the help of the Network Analyzer plug-in in Cytoscape software, the topology parameters of the network were calculated and analysed from the perspective of network node importance. Degree refers to the number of edges associated with a node. The greater the degree of a node is, the larger the node area in the graph. That is, the larger the node area is, the greater the importance of the node in the network. The compounds in Fig. 2 and their corresponding targets were used as network nodes. Figure 2 shows that one compound can act on multiple target genes, and multiple compounds can also act on one target gene at the same time. Among the compounds, MOL000422 (kaempferol), MOL000098 (quercetin), MOL003896 (7-Methoxy-2-methyl isoflavone), MOL004328 (naringenin), MOL000392 (formononetin) and MOL000358 (beta-sitosterol) occupied the largest area on the graph among all compounds and were important core compounds.

### Acquisition of disease target

A total of 251 genes related to COVID-19 were obtained by searching the Genecards database. The relevance score was used as the selection criterion to obtain the top 30 genes (see Table 2).

**Table 2:** Top 30 genes related to COVID-19

No	Gene symbol	Description	Relevance score
1	TNF	Tumor necrosis factor	33.08
2	IL6	Interleukin 6	31.28
3	CXCL8	C–X–C motif chemokine ligand 8	31.05
4	CD40LG	CD40 ligand	30.56
5	IL10	Interleukin 10	30.33
6	IFNG	Interferon gamma	27.48
7	CRP	C-Reactive protein	25.76
8	STAT1	Signal transducer and activator of transcription 1	22.73
9	MBL2	Mannose binding Lectin 2	22.1
10	TP53	Tumor protein P53	19
11	CCL2	C–C motif chemokine Ligand 2	18.13
12	IL2	Interleukin 2	17.68
13	CCL5	C–C motif chemokine Ligand 5	16.71
14	IFNA1	Interferon alpha 1	16.65
15	EGFR	Epidermal growth factor receptor	16.29
16	CXCL10	C–X–C motif chemokine ligand 10	15.3
17	TGFB1	Transforming growth factor beta 1	14.98
18	IL1B	Interleukin 1 beta	13.78
19	ACE2	Angiotensin I converting enzyme 2	12.32
20	CSF2	Colony stimulating factor 2	11.95
21	PPARG	Peroxisome proliferator Activated Receptor Gamma	11.93
22	CCR5	C–C motif chemokine Receptor 5 (Gene/Pseudogene)	11.37
23	CXCL9	C–X–C motif Chemokine Ligand 9	11.3
24	GPT	Glutamic–pyruvic Transaminase	11.12
25	MAPK1	Mitogen-activated Protein Kinase 1	11.09
26	CASP3	Caspase 3	10.88
27	IFNB1	Interferon beta 1	10.77
28	ALB	Albumin	10.68
29	FGF2	Fibroblast growth factor 2	10.53
30	SFTPD	Surfactant protein D	10.47

### Intersection of drug targets and disease targets

The above DYY drug target genes were intersected with COVID-19 disease targets to obtain possible genes associated with DYY treatment of COVID-19. The results showed that there was a total of 45 genes associated with DYY treatment of COVID-19 (see Additional file 2: Fig. S2).

### PPI analysis and core target screening

**Fig. 3:**
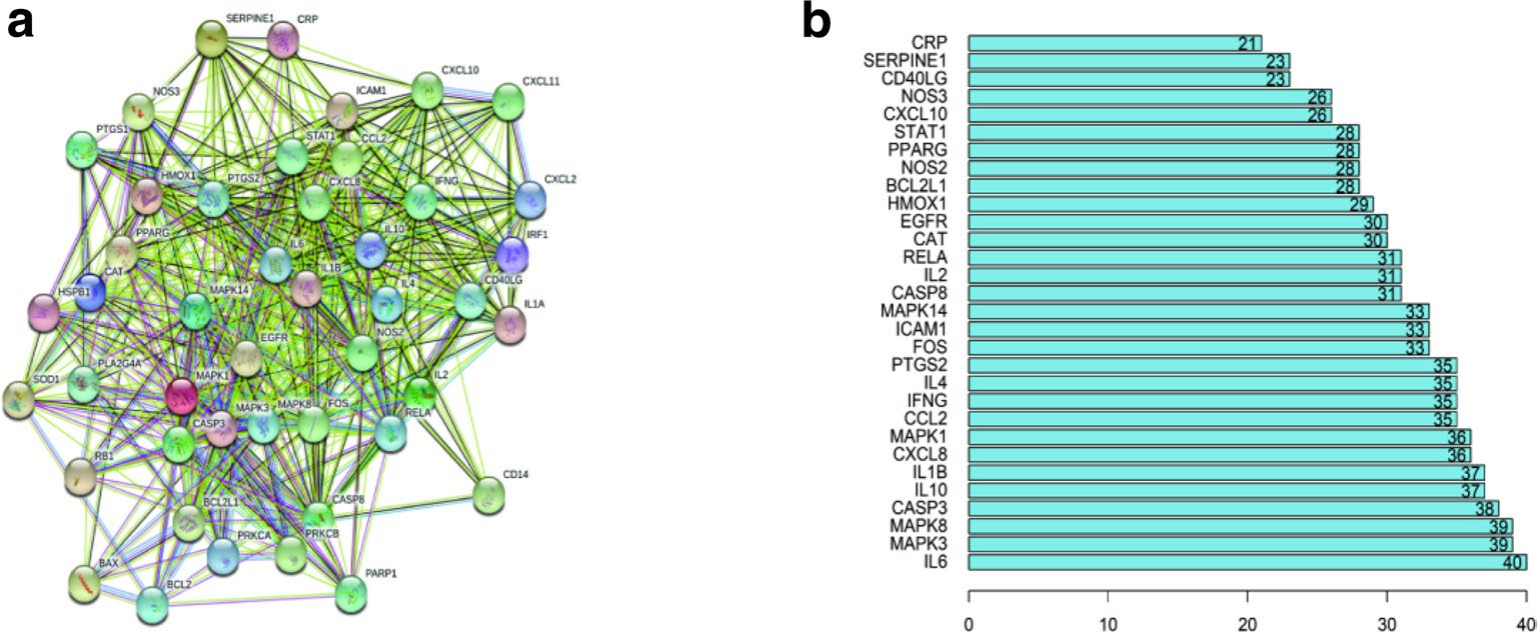
Protein interaction diagram and core gene bar chart. In the protein interaction diagram (**a**), the nodes represent proteins, and the connections represent interactions between proteins. The more connections there are, the greater the degree of connection. The node degree value indicates the number of connections between any node in the diagram and other nodes. In the core gene bar chart (**b**), the abscissa represents the number of genes, and the ordinate represents the name of the gene

The STRING database was used to draw a PPI network diagram of DYY for COVID-19 (see Fig. 3a). As shown in Fig. 3a, the network diagram consisted of 45 nodes and 581 edges, for which the average node degree was 25.8, and the PPI enrichment p-value was < 1.0e−16. The above PPI network was processed using R language, and the top 30 core genes were selected (see Fig. 3b). Figure 3b shows that the top 30 core genes had a node degree greater than 21, and the top genes, such as IL6, MAPK3, MAPK8, CASP3, IL10, IL1B, CXCL8, MAPK1, CCL2, IFNG and IL4, had a higher number of connections than other genes, all showing 35 or more connections.

### Construction of network visualization

**Fig. 4:**
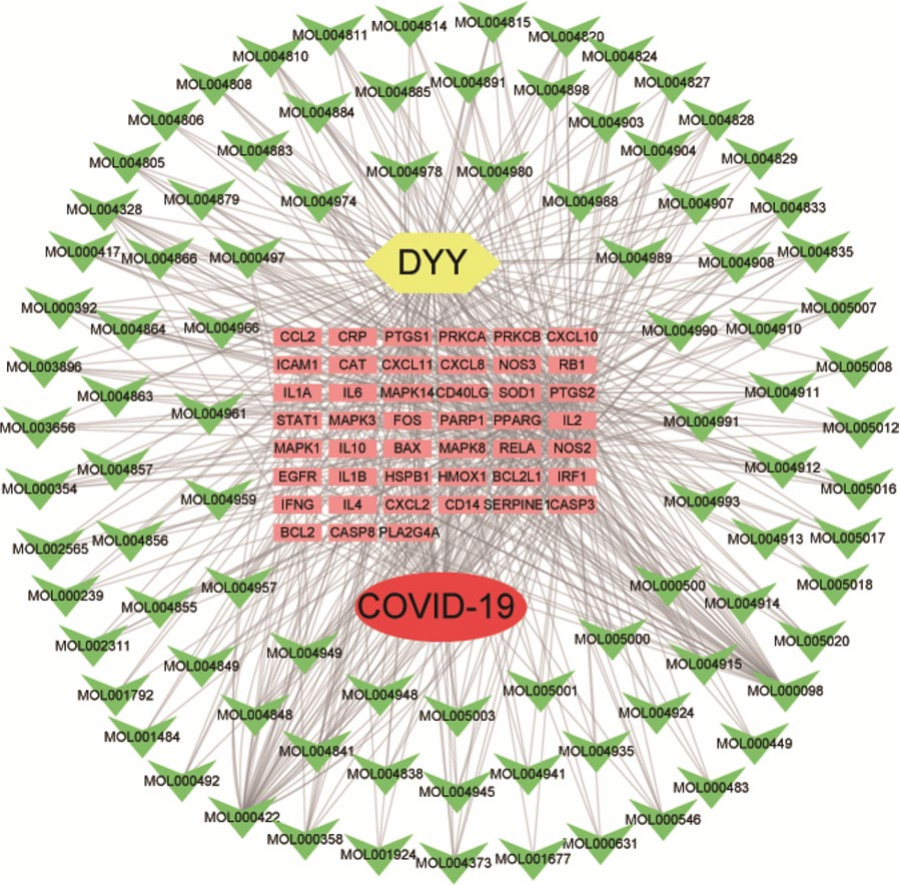
Drug-target-disease regulation network diagram. The yellow polygon in the figure represents the drug (DYY), the red circle represents the disease (COVID-19), the green arrows represent the compounds, and the pink boxes represent the targets of action

The active ingredients of DYY, the targets corresponding to the active ingredients, and the targets predicted for COVID-19 were imported into Cytoscape software to build a drug-target-disease network diagram (see Fig. 4). The network had a total of 139 nodes (including 94 compound nodes and 45 gene nodes) and 546 connections.

### GO analysis and KEGG pathway enrichment analysis

**Fig. 5:**
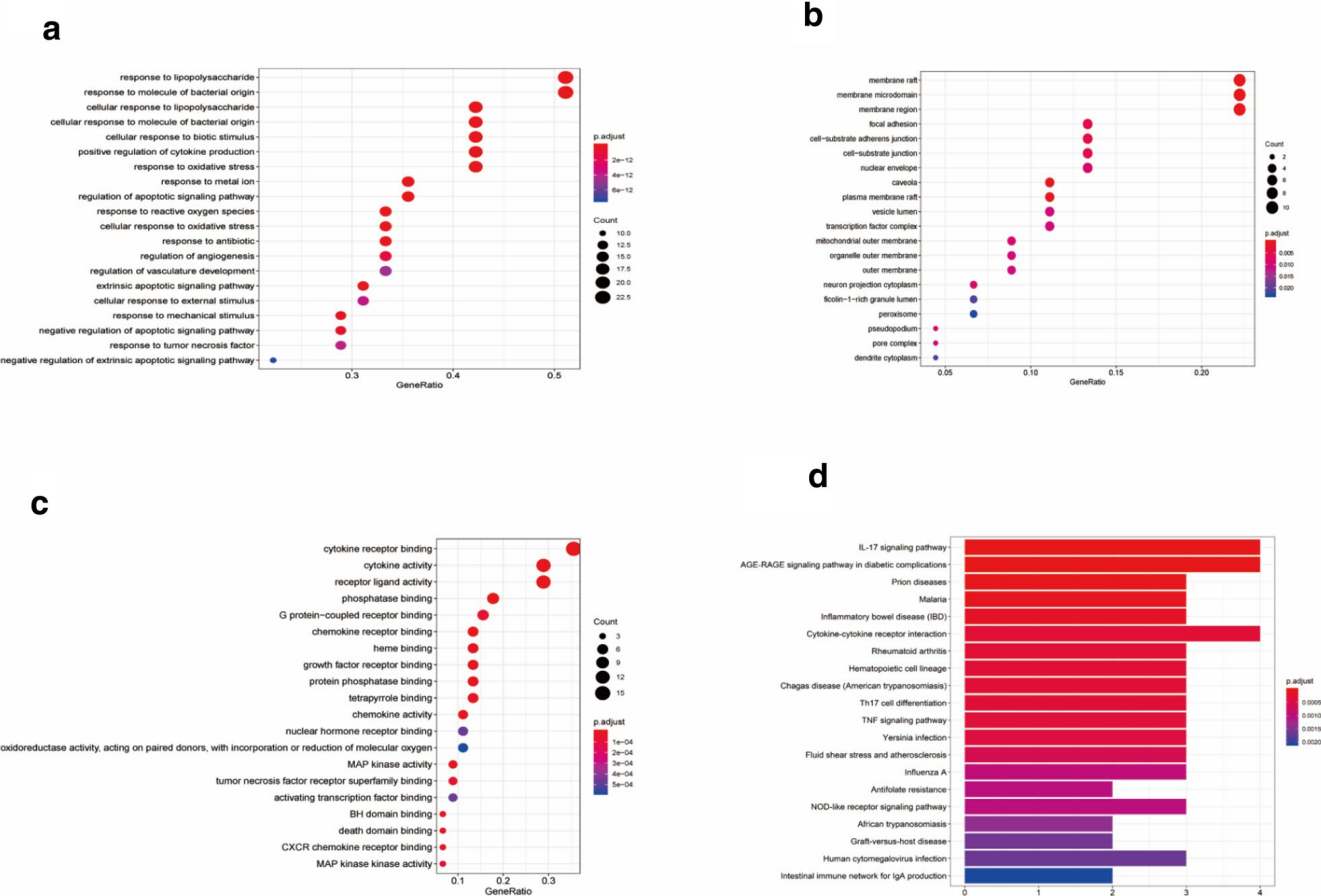
GO analysis and KEGG pathway enrichment analysis graphs. In the bubble charts in (**a–c**), the ordinate represents the names of the BP, CC, and MF terms, respectively, and the abscissa represents the degree of enrichment. In (**d**), the ordinate represents the names of the pathways, and the abscissa represents the number of genes enriched in the pathway. The P value indicates the significance of enrichment. The smaller the P value is, the higher the significance of enrichment, and the redder the colour on the graph

The R language and DAVID database were used for GO enrichment analysis by using the above-mentioned targets of DYY to treat COVID-19, and the number of biological process (BP), cellular component (CC), and molecular function (MF) entries was 1,506, 33 and 83, respectively. The top 30 biological processes were screened and are represented as graphical bubbles (see Fig. 5a–c). The KEGG pathway enrichment analysis identified 40 signaling pathways, and the top 20 entries were selected and are represented by a bar graph (see Fig. 5d).

### Construction of the KEGG relationship network

**Fig. 6:**
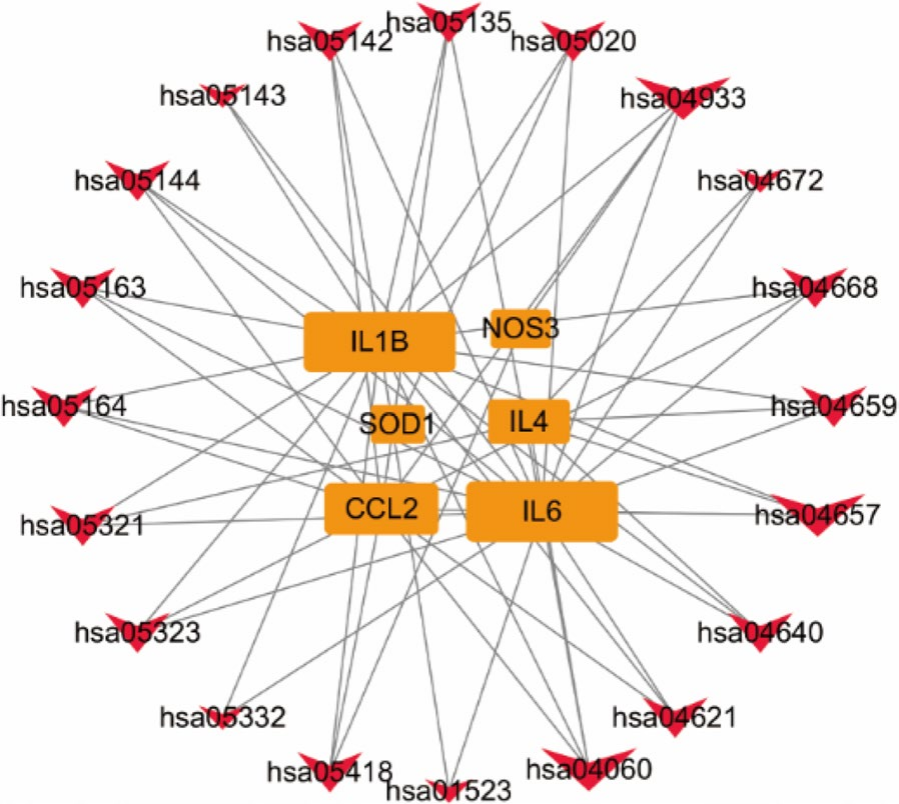
KEGG relationship network diagram. The red arrows indicate the pathway IDs. The larger the arrow is, the greater the number of genes connected to the pathway. The orange box indicates the name of the gene. The larger the box is, the greater the number of pathways connected

The top 20 pathways involved in DYY treatment of COVID-19 and the genes enriched in these pathways were imported into Cytoscape software to build a KEGG relationship network diagram (see Fig. 6). In Fig. 6, the pathways and the genes enriched in the pathways were used as network nodes, and we selected the top three pathways (hsa04657 (IL-17 signaling pathway), hsa04933 (AGE-RAGE signaling pathway in diabetic complications), and hsa0406 (cytokine–cytokine recev] ptor interaction pathway)) and top three target genes (IL6, ILIB, CCL2) enriched in these pathways according to degree.

### Molecular docking verification of core compounds and core target genes

The results obtained by the molecular docking software are shown in Table 3. The letters x, y and z were used to represent the size and position of the pocket. The final selected pocket is shown in bold in the column titled ‘Pocket size’. The results of the docking of the receptor and ligand are shown under ‘Docked complex’, and the residues docked with the small-molecule ligand are shown as yellow sticks. The structure with the initial ligand and the predicted protein pocket were processed by Discovery Studio 3.5 Client software, and the docked complex was processed by PyMOL software. As seen from Table 3, the scores for the five core compounds (kaempferol, quercetin, 7-Methoxy-2-methyl isoflavone, naringenin, formononetin) and protein crystal structures corresponding to the core target genes (IL6, IL1B, CCL2) were all greater than -5 kcal/mol, indicating that the compound had a certain affinity for the protein crystal structure. The interactions between some ligands (small-molecule compounds) and receptors (proteins) are shown in Additional file 3: Fig. S3. Additional file 3: Fig. S3 shows that the small-molecule compounds were tightly bound to the protein residues via various interactions.

**Table 3:**
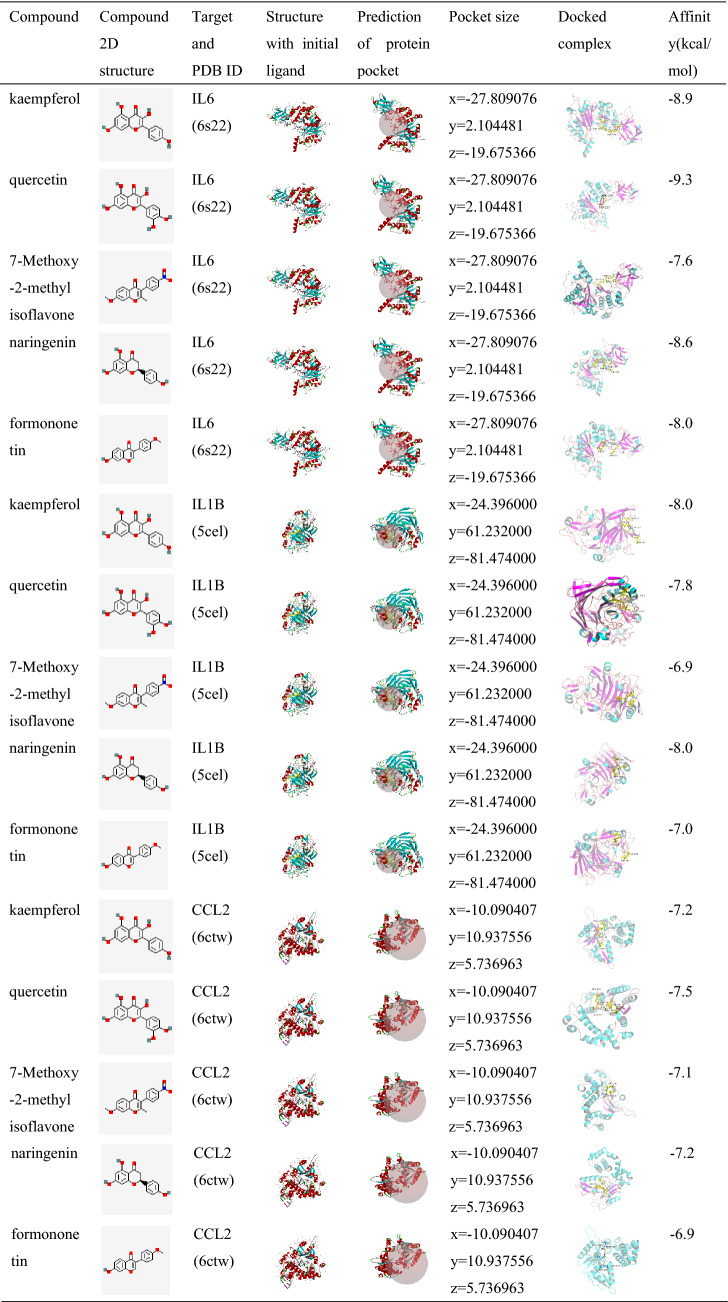
Molecular docking results

### Clinical validation of the core target IL6

**Fig. 7:**
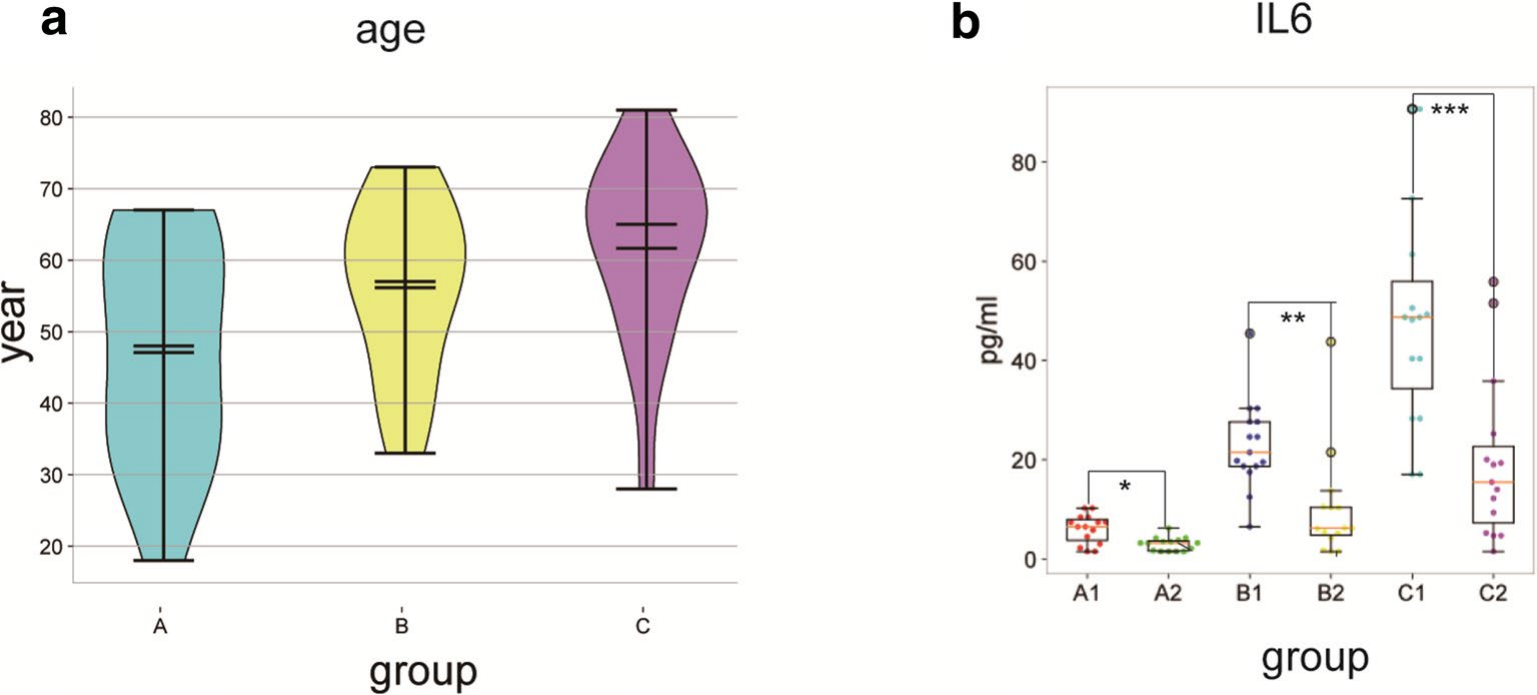
Graph of patient age distribution and IL 6 levels. In (**a**), the abscissa represents group, and the ordinate represents age. In (**b**), the abscissa represents the group, and the ordinate represents the level of IL6, where A1, B1, and C1 represent patients before treatment while A2, B2, and C2 represent patients after treatment in each group. Data are expressed as the mean ± S.D. (n = 15 per group); *p < 0.01, **p < 0.001, and***p < 0.001. the after treatment groups(A2, B2, C2) vs. the before treatment groups(A1, B1, C1)

Of the 45 patients selected, 15 were mild cases (group A), 15 were moderate cases (group B), and 15 were severe cases (group C). The age distribution of each group of patients and changes in IL6 levels before and after treatment are shown in Fig. 7a, b, respectively. Compared with the severe cases, Fig. 7a shows that the mild and moderate cases were younger. Figure 7b shows that a majority of the patients had different levels of IL6 elevation before treatment (the normal reference value of IL6 is 0–7 pg/ml), and the increase in IL6 was most pronounced in severe cases. After treatment, IL6 decreased in all groups, and differences within each group before and after treatment were statistically significant.

### Mechanism of action of DYY in the treatment of COVID-19

**Fig. 8:**
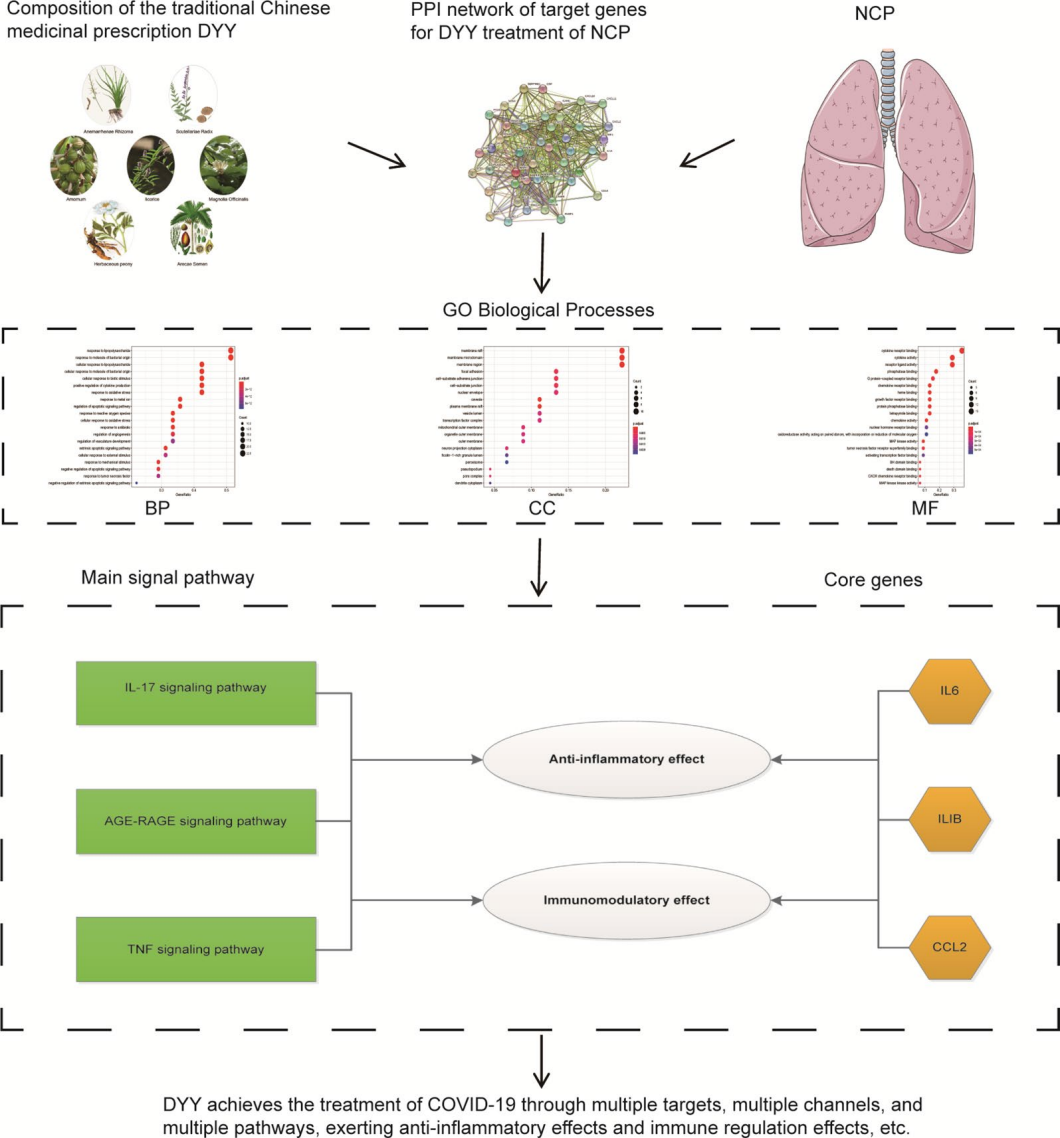
Mechanism of action of DYY in the treatment of COVID-19

Based on the above studies, the specific mechanism of the action of DYY in the treatment of COVID-19 is shown in Fig. 8.

## Discussion

As of April 6, 2020, the cumulative number of COVID-19 confirmed cases worldwide has exceeded 1.2 million. However, no vaccine or definitive antiviral drugs are available for the prevention and treatment of COVID-19. Therefore, it is crucial to find out medicines with confirmed curative effects for the prevention and treatment of COVID-19 as soon as possible to improve the patient’s condition and prevent the ongoing outbreak of COVID-19.

In this study, we first searched and screened a database of traditional Chinese medicine database to obtain 174 DYY compounds and 7053 corresponding target genes. Ent-epicatechin, quercetin, mairin, beta-sitosterol, sitosterol, and stigmasterol are common compounds of two or more Chinese medicines. Studies have shown that quercetin can reduce apoptosis induced by hypoxia and rescue phosphorylation of AMPK [15]. Beta-sitosterol has antipyretic, analgesic, anti-inflammatory, and antioxidant functions and plays roles in cough and phlegm elimination, immune regulation and tissue repair [16, 17]. Stigmasterol is present in the membrane [18] and has anti-inflammatory effects [19]. The compound-target network diagram (Fig. 2) shows that there was a complex network relationship between the compounds and the targets. Kaempferol, quercetin, 7-Methoxy-2-methyl isoflavone, naringenin, formononetin, and beta-sitosterol had the largest number of targets and were core compounds.

By searching the disease database, we found a total of 251 genes related to COVID-19. Drug target genes and disease related genes were intersected, and a total of 45 target genes for DYY treatment of COVID-19 were finally obtained (Additional file 2: Fig. S2). These genes were analysed by PPI analysis to obtain the corresponding network diagram (Fig. 3a). Figure 3a shows that the target genes of DYY for the treatment of COVID-19 were not independent, and there was a certain relationship among these genes. The core gene map in Fig. 3b shows that the top-ranking genes were IL6, MAPK3, MAPK8, CASP3, IL10, IL1B, CXCL8, MAPK1, CCL2, IFNG, IL4, etc. These genes were mainly concentrated in the inflammatory response, immune modulation, and cellular stress processes, which indicated that they might play a key role in DYY treatment of COVID-19. It is well known that IL6, IL10, IL1B, and IL4 are all members of the interleukin family. Interleukins play an important role in transmitting information, regulating immune cells, mediating T and B cell activation, and responding to inflammation [20, 21]. CCL2 and CXCL8 belong to the chemokine family and are important inflammatory cytokines. They play an important role in the migration of Tregs to inflammatory tissues [22] and in immune regulation in the body [23]. MAPK3, MAPK8, and MAPK1 are members of the MAPK family and can participate in responses to potentially harmful abiotic stress stimuli [24].

At the same time, a network of drug active ingredients, target genes corresponding to the active ingredients and disease targets was constructed as shown in Fig. 4. We can see that one compound can act on multiple target genes. Similarly, one target gene can also correspond to multiple compounds. That is, multiple compounds can act on a common target. Based on the above analysis, we concluded that multiple active ingredients in the traditional Chinese medicine prescription DYY can act on COVID-19 through multiple targets.

Through functional enrichment analysis of target genes for DYY treatment of COVID-19, GO biological process and KEGG pathway enrichment maps were obtained (see Fig. 5). It can be seen from Fig. 5 that in the GO terms, the BP terms (Fig. 5a) were mainly associated with the cell’s response to processes such as lipopolysaccharide, molecule of bacterial origin, biotic stimulus, cytokine production, oxidative stress, and adaptive signaling pathways. CC terms (Fig. 5b) were mainly associated with various membranes, including membrane raft, membrane microdomain, membrane region, plasma membrane raft, nuclear envelope, and mitochondrial outer membrane, etc.; the terms were also associated with focal adhesion, cell-substrate adherens junction, cell-substrate junction, etc. The MF terms (Fig. 5c) were mainly associated with various receptors (cytokine, chemokine, growth factor, CXCR chemokine, G protein-coupled, nuclear hormone receptors), binding functions (phosphatase, heme, protein phosphatase, tetrapyrrole, BH domain, death domain, tumor necrosis factor receptor superfamily) and various cytokine, ligand, and kinase activities (cytokine, receptor ligand, MAP kinase, chemokine, oxidoreductase). The pathways involved in the KEGG enrichment pathway (Fig. 5d) were mainly the IL-17 signaling pathway, AGE-RAGE signaling pathway, cytokine–cytokine receptor interaction, TNF signaling pathway, and NOD-like receptor signaling pathway. The diseases involved were mainly infectious and immune diseases. Infectious diseases included viral infectious diseases (prion diseases, influenza A, human cytomegalovirus infection, etc.), parasitic infectious diseases (malaria, Chagas disease, African trypanosomiasis, etc.) and bacterial infectious diseases (Yersinia infection). Immune diseases included inflammatory bowel disease, rheumatoid arthritis, and graft-versus-host disease.

As shown in Fig. 6, hsa04657 (IL-17 signaling pathway), hsa04933 (AGE-RAGE signaling pathway in diabetic complications), and hsa0406 (cytokine–cytokine receptor interaction pathway) enriched the highest number of genes, indicating that these pathways may play an important role in the mechanism of action of DYY in the treatment of COVID-19. The IL-17 signaling pathway is involved in the body’s immune response [25, 26] and inflammatory response [27]. The AGE-RAGE signaling pathway has important protective effects on bones and the heart and participates in oxidative stress response [25] and fibrosis transduction [22]. The cytokine–cytokine receptor interaction is a key pathway for regulating the cellular inflammatory response [28]. IL6, ILIB, CCL2 and other genes occupy a large rectangular area, indicating that there are more pathways connected to these genes. Therefore, it can be speculated that these genes play a key role in the mechanism of action of DYY in the treatment of COVID-19. IL6, ILIB, and CCL2 represent a wide range of inflammatory mediators and pathways. Many animal and human experiments have demonstrated that IL6 has a wide range of anti-inflammatory effects [29]. ILIB has analgesic, immunomodulatory, anti-hypoxia, and anti-inflammatory functions. CCL2 is an important inflammasome-associated chemokine [30]. Inhibition of CCL2 can reduce the infiltration of peripheral inflammatory cells such as monocytes and neutrophils [9]. NOS3 is a vasoprotective gene [31] that regulates vascular tone, blood pressure and platelet aggregation [32]. Research reports have shown that NOS3 can affect metabolism in the urea cycle of the methylation pathway, which is essential for preventing systemic inflammation [33].

By combining the core target gene bar chart (Fig. 3b) and the KEGG relationship network diagram (Fig. 6), we can see that IL6 is one of the most critical genes for anti-inflammatory and immune regulation in COVID-19 patients treated with DYY. Based on the comparison of COVID-19 patients before and after treatment with DYY, the IL6 level of COVID-19 patients increased to different degrees when they were admitted to the hospital but decreased after treatment, further confirming that DYY may play an important role in anti-inflammatory and immune regulation and may have other effects in the treatment of COVID-19 patients.

## Conclusions

In summary, we speculate that DYY may play an anti-inflammatory and immunoregulatory role in COVID-19 by acting on multiple target proteins, such as IL6, ILIB, and CCL2. The role of DYY involves a variety of biological processes, mainly signaling pathways such as the IL-17 signaling pathway, cytokine–cytokine receptor interaction, and AGE-RAGE signaling pathway, involved in diabetic complications. In short, DYY plays a role in COVID-19 treatment through multiple targets, multiple channels, and multiple pathways, making it worthy of clinical application and promotion. However, only part of the specific mechanism of action of DYY has been clinically verified, and further verification is needed in subsequent experiments.

## Supplementary information

**Additional file 1: Fig. S1.** Composition diagram of DYY.

**Additional file 2: Fig. S2.** Venn diagrams of drug targets and disease targets.

**Additional file 3: Fig. S3.** Two-dimensional structure diagram of ligand-receptor interaction. The interaction forces between the small-molecule compound ligands and protein receptors in Fig. S3 are shown in different colors. Purple to gray represent electrostatic, van der waals, convalent bond, water and metal interaction, respectively.

## Data Availability

The data used to support the results of this study can be obtained from the first author upon reasonable request.

## Abbreviations

COVID-19: Coronavirus disease 2019
DYY: Dayuanyin
TCMSP: Traditional Chinese Medicine Systems Pharmacology Database and Analysis Platform
DL: Drug-like
OB: Oral bioavailability
BBB: Blood–brain barrier
PPI: Protein–protein interaction
GO: Gene Ontology
KEGG: Kyoto Encyclopedia of Genes and Genomes
MO: Magnolia officinalis
AM: Amomum
AS: Arecae semen
HP: Herbaceous peony
SR: Scutellariae radix
AR: Anemarrhenae rhizoma
LR: Licorice
BP: Biological process
CC: Cellular component
MF: Molecular function
