# Supplementary figures and images for: Mechanism of Dayuanyin in the treatment of coronavirus disease 2019 based on network pharmacology and molecular docking
Source: Chin Med. 2020 Jun 12;15:62. doi: 10.1186/s13020-020-00346-6 (PMC7289712; doi:10.1186/s13020-020-00346-6)

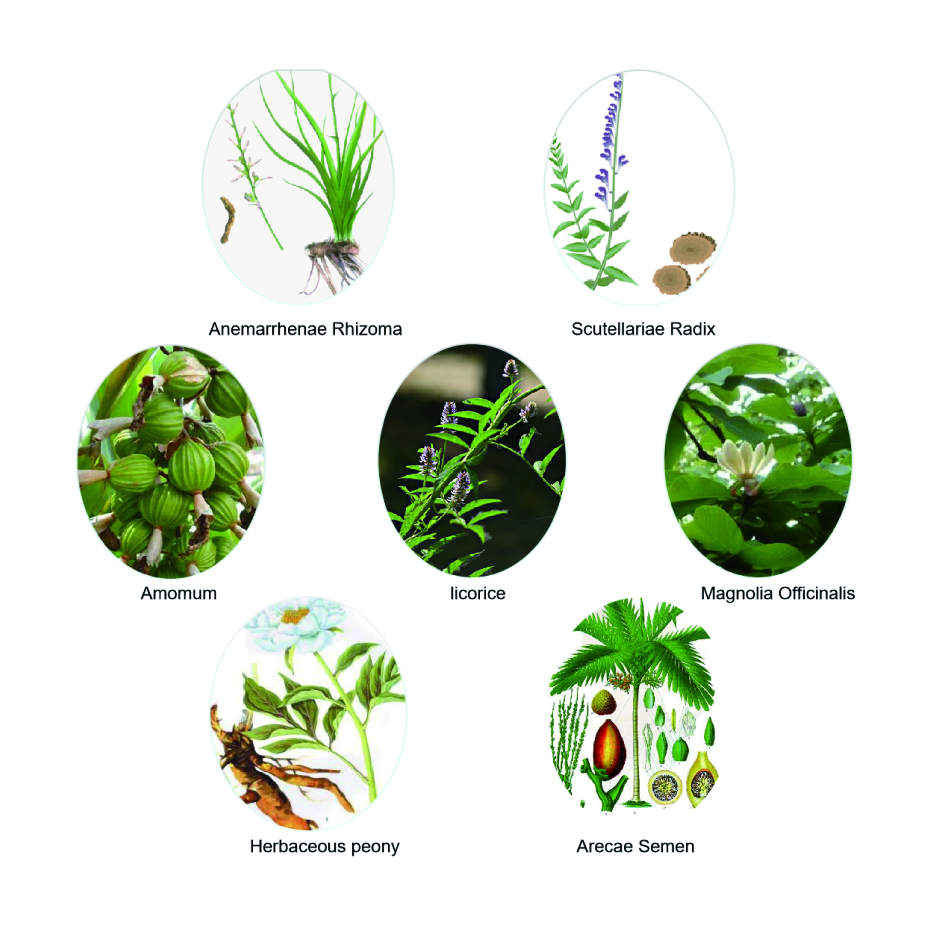

Supplement: Supplementary file 1 — Additional file 1: Fig. S1. Composition diagram of DYY. [file 13020_2020_346_MOESM1_ESM.tif]

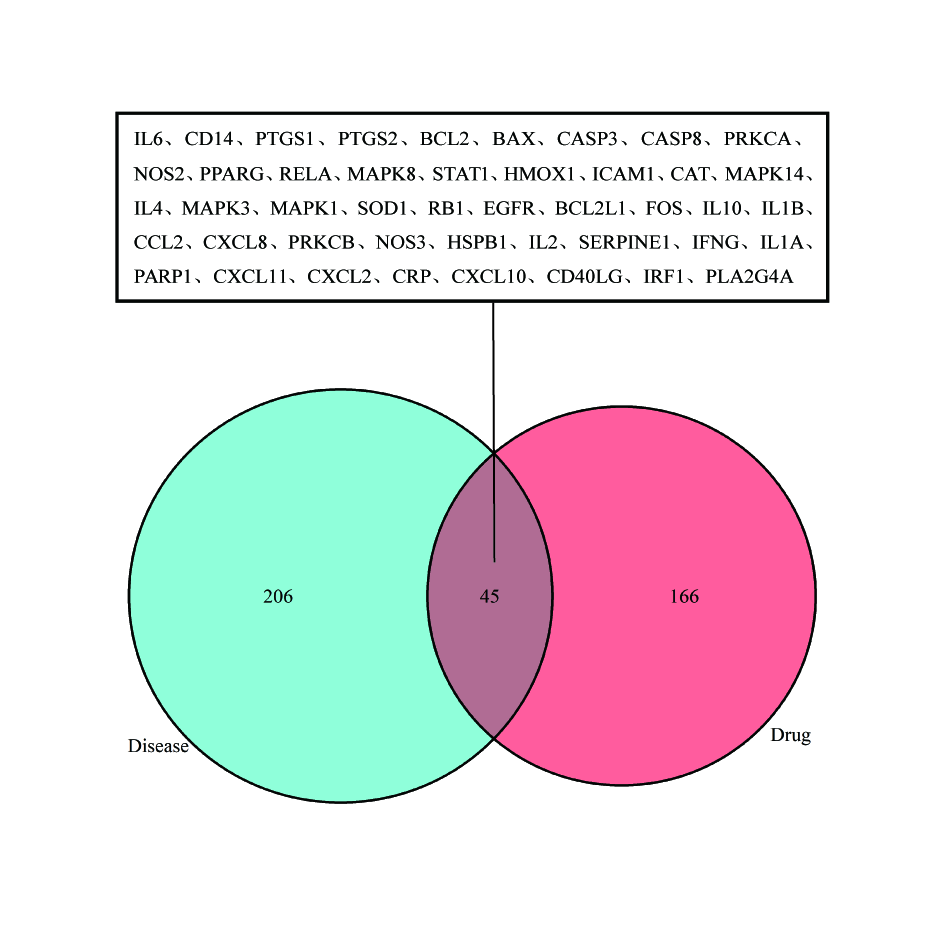

Supplement: Supplementary file 2 — Additional file 2: Fig. S2. Venn diagrams of drug targets and disease targets. [file 13020_2020_346_MOESM2_ESM.tif]

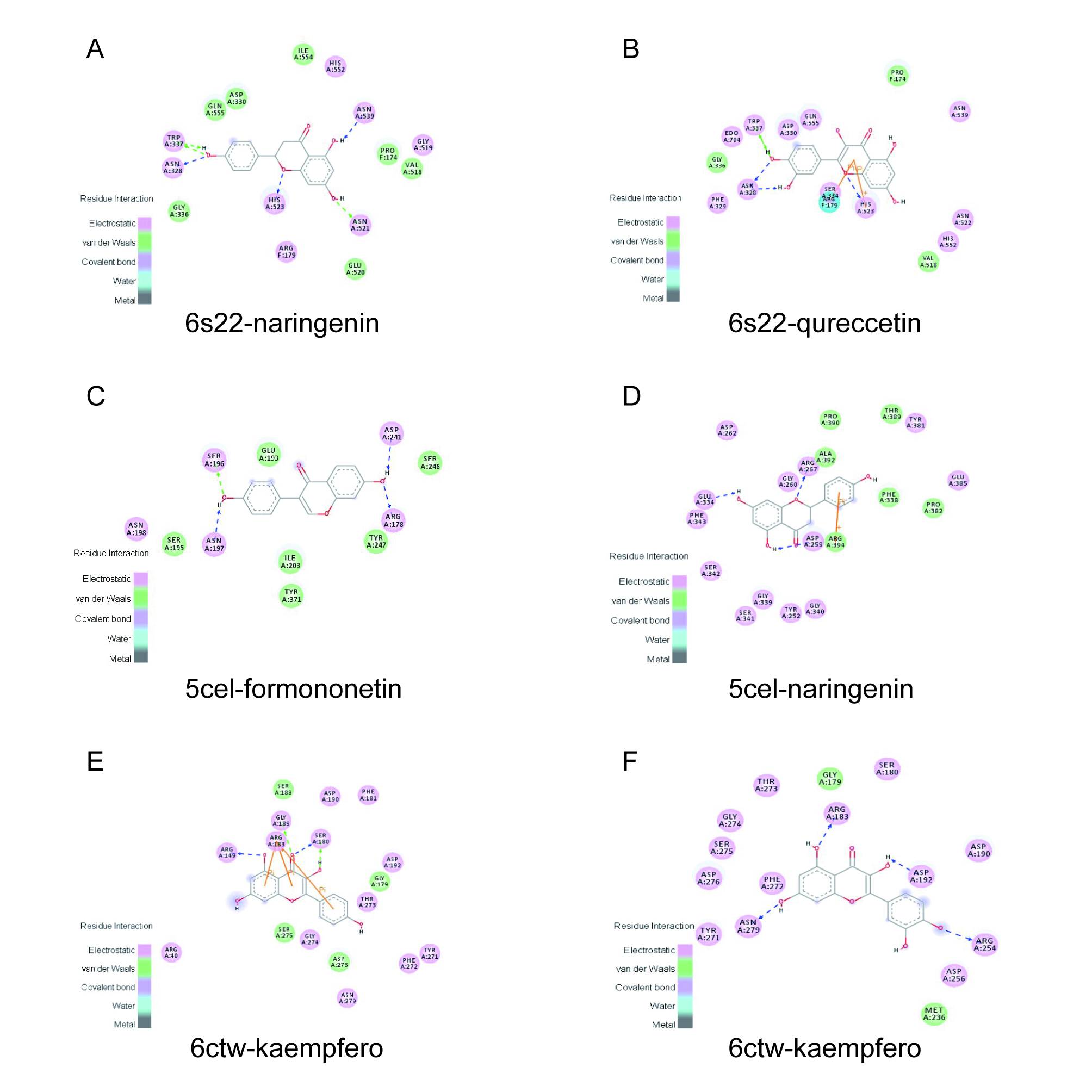

Supplement: Supplementary file 3 — Additional file 3: Fig. S3. Two-dimensional structure diagram of ligand-receptor interaction. The interaction forces between the small-molecule compound ligands and protein receptors in Fig. S3 are shown in different colors. Purple to gray represent electrostatic, van der waals, convalent bond, water and metal interaction, respectively. [file 13020_2020_346_MOESM3_ESM.tif]
